# Appraising the risk level of physicochemical and bacteriological twin contaminants of water resources in part of the western Niger Delta region

**DOI:** 10.1007/s10661-020-08302-5

**Published:** 2020-05-03

**Authors:** Azuka Ocheli, Onyeka Benjamin Otuya, Star Otitie Umayah

**Affiliations:** 1 Department of Geological Sciences, Nnamdi Azikiwe University, Awka, Anambra State Nigeria; 2 Department of Chemical Sciences, Novena University, Ogume, Delta State Nigeria; 3 Department of Geology, Delta State University, Abraka, Delta State Nigeria

**Keywords:** Physicochemical, Bacteriological, Waterborne diseases, Contaminants, Water quality

## Abstract

This study was carried out to assess the physicochemical and bacteriological contaminants of surface, shallow well and municipal borehole waters in part of the western Niger Delta as a way of safeguarding public health against waterborne diseases. A total of 72 water samples collected from the study area were analysed and their results show that the pH average value ranges from 6.2 in the dry to 8.5 in the rainy seasons for surface water, 6.6 and 8.3 for shallow well water and 6.5 to 8.4 for borehole water. The turbidity in surface water varies from 8.1 to 26.2 Formazin Attenuation Unit (FAU), 0.3 to 2.9 FAU in shallow well water and 0.4 to 4.8 FAU in borehole water. Electrical conductivity (EC) value varies from 1010 to 1840 μs/cm for surface water, 201 to 950 μs/cm for shallow well water and 670 to 1650 μs/cm for borehole water. Total dissolved solids range from 0.1 to 2.6 mg/l in surface water, 0.2 to 4.1 mg/l in shallow well water and 0.3 to 4.4 mg/l in borehole water. The value of the total hardness ranges from 44 to 120 mg in surface water, 46 to 96 mg in shallow well water and 70 to 130 mg in borehole water. The nitrate value ranges from 1.1 to 10.6 mg in surface water, 3.0 to 8.4 mg in shallow well water and 3.7 to 9.6 mg in borehole water. The value of sulphate content varies from 4.6 to 38.5 mg in surface water, 6.2 to 34.8 mg in shallow well water and 5.7 to 55.7 mg in borehole water. The value of phosphate concentrations in surface water varies from 1.0 to 9.0 mg, 0.7 to 3.4 mg in shallow well water and 1.0 to 4.7 mg in borehole water. The bacteriological analysis using the membrane filtration technique revealed the presence of faecal bacteria and total coliform counts. The presence of the analysed twin contaminants in the studied water resources reduced their water quality. The physicochemical and bacteriological data were subjected to statistical and correlation tests. It was concluded that their concentration levels were independent of intra-seasonal changes. The likely natural and artificial sources of contaminants are run-offs from fertilized lands, septic tanks, industrial discharges, sewage and waste disposal, algae blooms and erosion from natural deposits. The inherent risk is water-related diseases such as waterborne diseases, water-washed diseases, water-based diseases and diseases transmitted by water-related insect vectors. Water resources should be protected through proper sanitation systems, limiting of up-stream discharges, maintenance of wellheads, boiling and municipal water treatment plants. Educational advice should be given to the inhabitants on the dangers posed by continuous drinking of contaminated water. These measures will prevent disease outbreak and public health burden in the area.

## Introduction

The inhabitants in the cities and rural areas in part of the western Niger Delta, Nigeria, are not adequately supplied with water of good quality by federal and state governments to meet the ever-increasing clean water demand which is indispensable to man’s continued existence. These inhabitants are therefore forced to use water from the surface, shallow wells of depth below 50 ft and municipal boreholes for domestic, agricultural and industrial purposes which might have the possibility of being contaminated through natural and anthropogenic source derivatives. Surface water in the form of ponds, rivers, lakes, streams provides valuable palatable water that is used for the well-being of humanity. Rivers are immensely important due to tremendous water holding and large perforated capillary network which confirms the annual freshwater availability (Jayalakshmi et al. 2011). Natural sources of water resources contaminants are rock types, sediments, migratory pathways (Hong et al. 2015) and seasonal variations (Trivede et al. 2010). Activities of man contaminate surface and sub-surface water sources through inadequate source protection, untreated waste disposal, dissemination of chemicals and micro-bacteriological matter, leakages, washings, run-off from poorly constructed drainage along the roads, waterway transportations and chemicals used in agricultural production such as fertilizers, pesticides and herbicides. These chemicals accumulate and migrate to the water tables thereby deteriorating the physical, biological and chemical water quality. Septic tanks, pit/open latrines, petrol and gas stations as well as refineries built close to the surface, hand-dug wells and municipal boreholes as well as the proximity of some boreholes to solid waste and chemical dumpsites contaminate both surface and sub-surface water. Origin (headwater), environmental mediums and receptors are the three fundamental elements in environmental threat (Yao et al. 2015) that lead to environmental consequences and adversely affect humans and the economy (Yao et al. 2015). The hydrogeochemistry of groundwater in any geological formation in an environment is structured by the chemistry of the percolated water at the recharge resource, the chemistry of the porous and pervious mediums including the cementing materials, the matrix of the aquifer, rate of groundwater flow and the travel time of the water via the environment (Omoboriowo et al. 2012). Impacts of a solid waste disposal site on soil, surface water and groundwater quality in Dar-es-Salaam, Tanzania, was studied by Kessenga and Mbuligwe (2009), and they found out that higher faecal coliform counts are in sites downgradient of the study area. Also, from the study on groundwater contamination in Epworth, Zimbabwe, by Chidavaenzi et al. (2000), they observed that groundwater contamination was higher in the dry season than the wet season. They also confirmed that coliforms are always detected about 20 m away from the pit latrines. An assessment of heavy metal concentrations in hydraulically connected water systems in the Northern Delta Depobelt, Nigeria, has been carried out by (Ocheli et al. 2018). It was observed that Zn, Cd, Cu, As and Cr concentrations were below the recommended toxic level by the World Health Organization (WHO) for safe drinking except for Fe and Pb. A comparative analysis of zinc (Zn) and lead (Pb) concentrations in sediment samples from the traditional hand-dug wells in Umukwata and its environs, Delta State, Nigeria, revealed that Zn and Pb concentrations were below effect range median (ERM) and uncontaminated status. It was recommended that regular monitoring should be done to avoid bio-accumulation in the future (Ocheli et al. 2017). Physicochemical and bacteriological quality assessment of hand-dug well waters in Igarra town, Edo State, Nigeria, has been carried out by (Osayande et al. 2015). It was observed that the water samples were of poor microbiological standards and also had a high concentration of iron and zinc. Bacteriological and physicochemical examination of well waters in Ahmadu Bello University (main campus), Zaria, Nigeria, revealed that the number of faecal coliforms was higher than the recommended standard (Okuofu et al. 1990).

Poor funding, shortage of facilities, poor water resource management, corrupt practices by government officials and contractors as well as political instability are the major challenges in the provision of good quality water in Nigeria in recent years and have continued to impede water development in Nigeria. Water planners, managers, individuals and governments always put more pressure on only water quantity and outright neglect of water quality. Water supplies in the rural areas and most urban cities are supplied to the people without treatment before use for domestic, agriculture and industrial purposes. Where treatments are done, during floods in the raining season, sediments are flocculated and removed periodically. Borehole waters are rarely treated and are sometimes supplied to the homes directly from the aquifers. The poor quality water supplied is the major causes of waterborne diseases particularly typhoid fever and cholera (Okuofu et al. 1990). The hydrological cycle is a blatant mode of spreading enteric diseases marked by intestinal inflammation and ulceration. According to Hutchinson and Ridgeway (1975) and Okuofu et al. (1990), bacteriologically polluted water is potentially dangerous to health because of possible outbreaks of typhoid, cholera epidemics. Also, poor physicochemical quality may have adverse health effects causing avoidable economic and human losses (Hutchinson and Ridgeway 1975; Okuofu et al. 1990). It has been revealed by several workers notably (Hutchinson and Ridgeway 1975; Okuofu et al. 1990; Osayande et al. 2015; Yao et al. 2015) that there is a direct correlation between health standards and regression of many endemic diseases and adequacy or otherwise of potable drinking water or domestic purposes. Water quality guidelines by World Health Organization (WHO) and Standards Organization of Nigeria (SON) and Federal Ministry of Environment (FME) have been used for appraising the acceptability of public drinking water supplies (Okuofu et al. 1990; Osayande et al. 2015; Ocheli et al. 2017, 2018). This study focuses on the physicochemical and micro-bacteriological twin water contaminant appraisal to get detailed information on the quality of water in the study area, documentation on the consequences of the presence of contaminants in water for drinking and domestic purposes and recommendation of strategies for improving the water quality.

### Location and accessibility

**Fig. 1:**
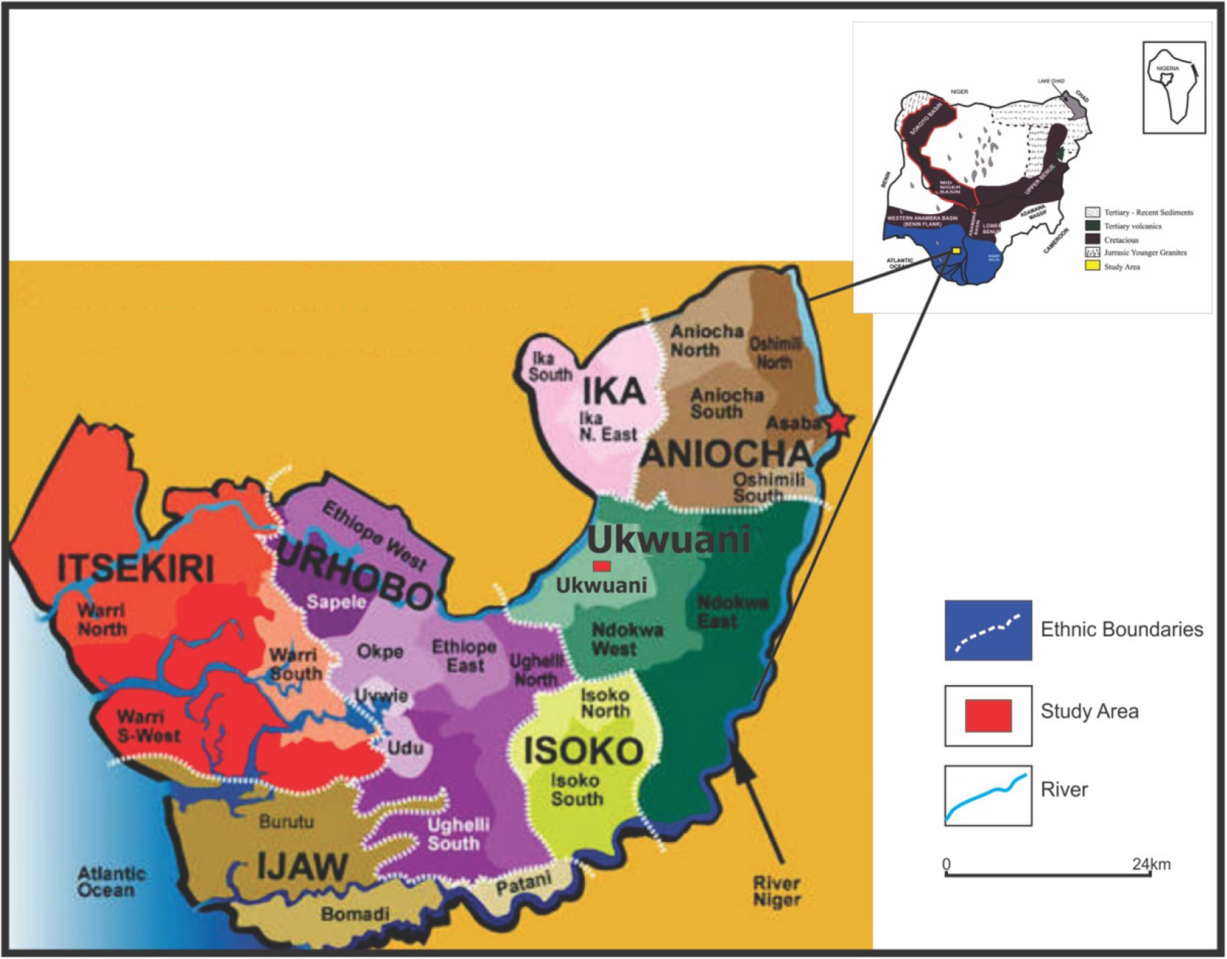
Map of Delta State showing the study area (modified from globalsecurity.org; Obaje et al. 2004; Ocheli et al. 2017)

**Fig. 2:**
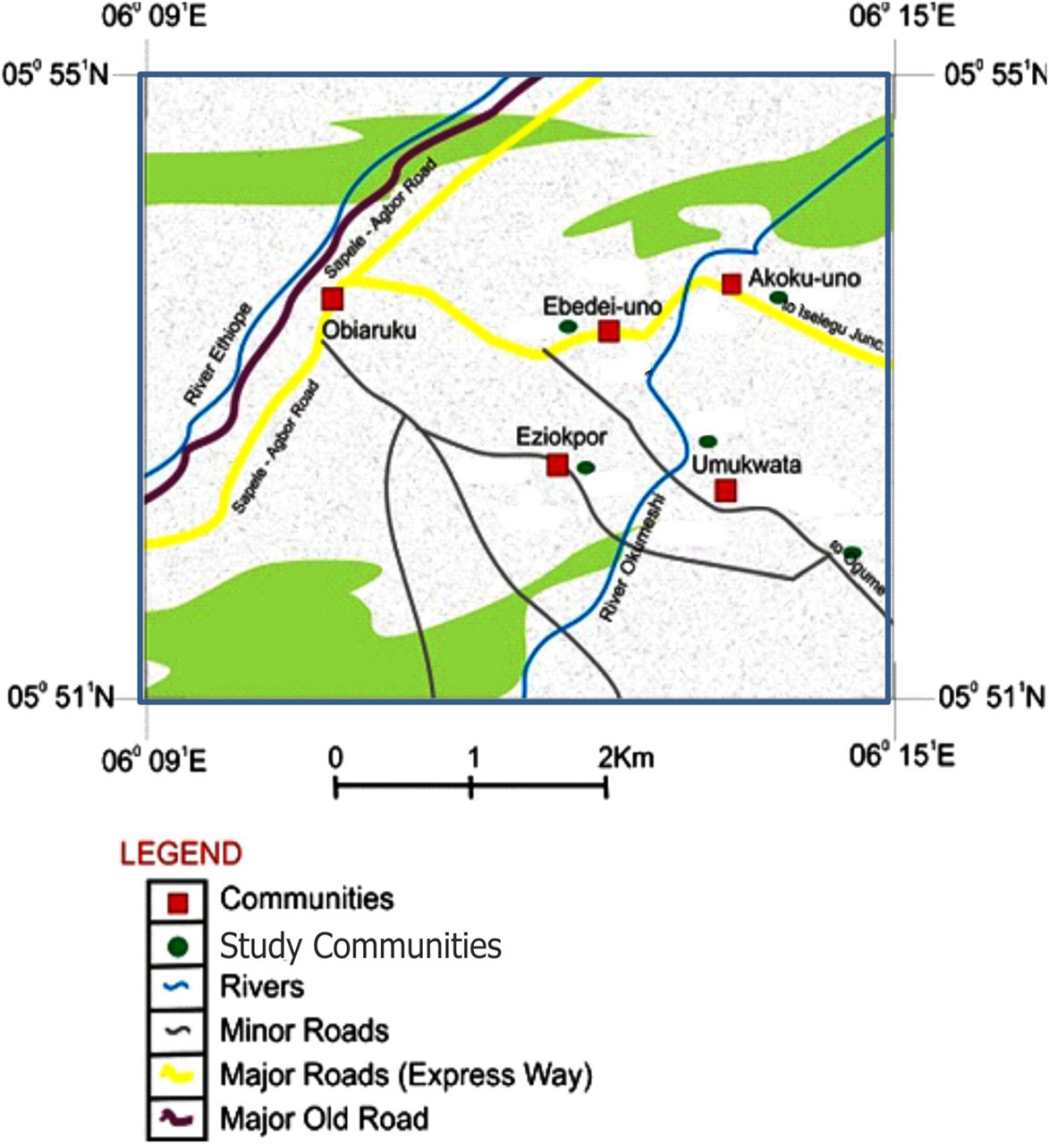
Map showing the study communities (Ocheli et al. 2017, 2018)

The four communities of study within Ukwuani Local Government Area of Delta State, Nigeria (Fig. 1), are typical rural communities, about 124.5 km from the state headquarters, Asaba-Agbor-Obiaruku route. It lies between latitude 05° 51′ N to 05° 55′ N and longitude 06° 09′ E to 06° 15′ E (Fig. 2). The terrain is undulating with thick forest along the low river valley and light savannah. The study is transversed by its major road such as Obiaruku-Utagba-Uno road and minor roads such as Obiaruku-Eziokpor-Umukwata-Ebedie roads which made accessibility easier to collect water samples for laboratory analysis. The communities were drained by river Okumeshi, sourced from the Utagba-Uno area and flows through freshwater swamps, mangrove swamps and coastal sand ridges which empty into brackish Farcado that in turn empties into the Atlantic Ocean. The topography and the geology placed the four communities within the tropical wet belt where farming lands support tropical crops most especially during the raining season. The rain begins from April to October which serves as the planting season while November to March is a relatively dry season and it serves as the harvest season.

### Geological setting of the study area

Several workers have published detailed information on the geological setting of the study area (Allen 1965; Oseji and Ofomola 2010). The communities fall within the oil-rich region of the Niger Delta province, Nigeria. The sub-surface geology comprises of three lithostratigraphic units ranging from the Akata Formation at the bottom, the Agbada Formation at the middle and the Benin Formation at the top (Allen 1965; Short and Stauble 1967; Avbovbo 1978; Evamy et al., 1978; Doust and Omatsola 1990; Oseji and Ofomola 2010; Ogbe et al., 2013; Ocheli et al. 2017, 2018). The topmost quaternary deposits within the study area are classified to be coastal plain sands, sambreiro, Warri deltaic plain deposits encapsulated by mangrove, wooded back swamps, freshwater swamp and meander belts (Allen 1965; Oseji and Ofomola 2010; Ocheli et al. 2017, 2018).

## Materials and methods

### Water sampling and collections

Water samples were collected from twelve (12) surface water points, twelve (12) hand-dug wells and twelve (12) municipal boreholes located in four communities: Akoku-Uno, Ebedi-Uno, Eziokpor and Umukwata (Fig. 2) where oil exploration and exploitations are currently ongoing as well as agricultural activities. Sampling was done during the dry and rainy seasons. Water sampling and collections were done in duplicate to minimize systematic error. Water samples were drawn with the aid of a sterile 500-ml screw-capped bottle drawer for hand-dug wells and rivers. Water samples from the boreholes were collected by pumping out the water directly into the sterilized bottles through the purging process. The bottles were tightly corked after filling, well labelled and immediately preserved in a lightproof insulated box containing ice packs for rapid cooling. The water samples were conveyed to the laboratory and kept at 4 °C before the time of physicochemical and bacteriological analyses. The time between the sample collection and analysis ranges between 4 and 6 h. The colours, tastes and odours of the water were noted during sampling and collections.

### Physicochemical analysis

Physicochemical parameters determined in water sampled in the study area were pH, turbidity, electrical conductivity, total dissolved solids, total hardness, nitrate, sulphate and phosphate. The pH and electrical conductivities were determined using a digital pH meter (model GMBH D4040 NEUSSI) and a conductivity meter (Radiometer Copenhagen CDM83). The turbidity of the samples was done using gravimetric standard procedure described by Ademoroti (1996) and adapted by Osayande et al. (2015). The total hardness and presence of sulphate of the water samples were determined using titrimetric procedures described by Ademoroti (1996) and adapted by Osayande et al. (2015). The concentration of nitrate and phosphate were determined by UV/VIS spectrophotometer at 410 nm using ethylenediaminetetraacetic acid (EDTA) as described by American Public Health Association (APHA 2005) and the ascorbic acid reduction methodology described by American Society for Testing and Materials (ASTM 1990). The physicochemical data were subjected to statistical and correlation tests.

### Bacteriological analysis

Water samples were analysed for the presence of total coliforms and faecal coliform counts (*Escherichia coli*) by membrane filtration technique as described by the United States Environmental Protection Agency (USEPA 2009) using 0.45-μm cellulose acetate membrane filters. After 10 ml of the water, samples were thoroughly mixed, dilutions of 10–1 and 10–2 were done, and a vacuum was applied and then filtered each time at 20 m/s using a glass membrane filter funnel which was sterilized by flaming with methylated spirit. The inoculation and incubation of plates were done by following the procedures reported by Okuofu et al. (1990). Membrane filters were hygienically rolled on the surface of the required media using Millipore forceps. All plates were wrapped in cellophane paper to prevent drying of the media and incubated for 4 h at a temperature of 35 °C. Subsequently, they were transferred to an incubator at a temperature of 44.5 °C. The reason for temperature difference is that normal coliforms are capable of growth only at 35–37 °C while thermotolerant coliforms such as *E. coli* possess B-galactosidase (Edberg et al. 2000) which only give provisional acid production and ferment lactose and confirmation by the growth and fermentation of lactose at 44.5 °C. Membrane-enriched Teepol Broth (METB) plates were left for 18–20 h at this temperature while Sianetz and Barthly Medium (SBM) plates were left for 40–44 h. The incubation facilitates the growth of the organisms in the form of colonies on the upper surface of the membrane. This is because they become acclimatized to the new conditions. Following this period of incubation, all yellow colonies on filters incubated on METB were isolated and counted as *E. coli* on adsorbent pads saturated with the METB. All red-pink colonies were counted as faecal coliforms on SBM. The resulting coliform cultures were further screened for characters of bacteria and identification by carrying out confirmatory biochemical tests described by (Cullimore 2000) and the results compared with the identification charts of (Akeredolu 1991; Cullimore 2000). *Escherichia coli* (*E. coli*) were detected by adding few drops of Kovac reagent to each of the MTEB. The yellow colour of the reagent changes to pinkish-red on the paper dipped into MTEB indicating the presence of *E. coli*. Also, acid and gas production in the SBM indicates the presence of faecal coliforms. The bacteriological data were subjected to statistical and correlation tests.

## Results and discussion

### Results of the physicochemical analysis

The results of the physicochemical parameters of water samples from the surface, shallow well and municipal borehole in the four communities of the study area which vary from dry to rainy seasons are presented in Tables 1 and 2. The mean pH value ranges between 6.2 and 8.5 for surface water, 6.6 and 8.3 for shallow well water and 6.5 to 8.4 for borehole water. pH is a measure of hydrogen-ion concentration in the water. Water with a low pH is more acidic, while water with a higher pH is harder or more alkaline. It indicates that the water samples from the area range from slightly acidic to alkaline. The pH of the water is very significant because most activities can occur only within a short range; thus, any deviation from the acceptable limit could be deadly to a particular microorganism. Osayande et al. (2015) reported that there was no potential risk arising from the consumption of either highly acidic or alkaline water but the fact remains that people with gastritis and gastrointestinal problems such as *Helicobacter pylori* are associated with those that consume acidic water. (USEPA 2019) recommends that suitable water for drinking has a pH between 5.5 and 9.0. All water samples collected from the study area had pH within the recommended standard by WHO, SON (2007) and (USEPA, 2019). Therefore, the water in the study area is judged to be suitable for drinking based on the pH. The turbidity in surface water varies from 8.1 to 26.2 Formazin Attenuation Unit (FAU), 0.3 to 2.9 FAU in shallow well water and 0.4 to 4.8 FAU in borehole water. Turbidity results for shallow well water were within the (WHO, 1997; 2005; 2008; SON, 2007) standard for drinking except that of water from some of the borehole and surface waters (river and ponds) that exceeded 5.0 FAU. The presence of particulate matter such as clay, silt, suspended finely divided organic matter or the microscopic organisms’ leads to a reduction in transparency (Osayande et al. 2015). The reason for high turbidity in borehole water could be attributed to the corrosion of the pipes resulting from the use of the hand pump (Ibe et al. 2002). Corrosion of hand pump allows soil particles to seep into the water thereby causing high turbidity. The surface water in the study area is generally black, resulting from the soil, rock types and the entire geological formation. Clean water is colourless, tasteless and odourless in an ideal situation. The colloidal materials provide adsorption sites for the chemical that are dangerous to health or cause undesirable tastes or odour (Qing, 2016). Also, the presence of organic substances from the discharge of water from cassava processing industries results to odour as well as indicative of microbial activity. The turbidity and coloured water from the surface, shallow well and borehole reduce the water quality in the study area. Turbidity and colour can as well vary with contamination of the bacteria which degrade the nutrients in the water. Electrical conductivity (EC) is related to the measurement of water’s current and is directly related to the concentrations of the ionized substance in the water (Jayalakshmi et al. 2011, Osayande et al. 2015). The EC value varies from 1010 to 1840 μs/cm for surface water, 201 to 950 μs/cm for shallow well water and 670 to 1650 μs/cm for borehole water. The EC values greater than 200 μs/cm indicate the presence of ionized substances in all the water samples from the study area, confirmed by the presence of sulphate and nitrates anions (Tables 1 and 2). Total dissolved solids range from 0.1 to 2.6 mg/l in surface water, 0.2 to 4.1 mg/l in shallow well water and 0.3 to 4.4 mg/l for borehole water. All the water samples in the study area are below the recommended lower limit standards set by the WHO and SON. Total hardness (TH) is a measure of the capacity of water to precipitate soap mainly by calcium and magnesium ions. The value of the total hardness ranges from 44 to 120 mg in surface water, 46 to 96 mg in shallow well water and 70 to 130 mg in borehole water (Tables 1 and 2). All the water samples from the study area are of good water quality based on total hardness content as they are below the recommended lower limits for drinking water set by the WHO, SON and FME. Tables 1 and 2 reveal that nitrate value ranges from 1.1 to 10.6 mg in surface water, 3.0 to 8.4 mg in shallow well water and 3.7 to 9.6 mg in borehole water. The value of sulphate content varies from 4.6 to 38.5 mg in surface water, 6.2 to 34.8 mg in shallow well water and 5.7 to 55.7 mg in borehole water. The value of phosphate concentrations in surface water varies from 1.0 to 9.0 mg, 0.7 to 3.4 mg in shallow well water and 1.0 to 4.7 mg in borehole water. The nitrate, sulphate and phosphate levels were below the standard limits set for portable water by (WHO, 1997; 2005; 2008; SON, 2007; FME, 1996; 2001), but the presence of sulphur in drinking water causes noticeable taste and therefore reduces its water quality. The main sources of nitrate in groundwater could be attributed to agricultural practices such as the application of nitrogenous fertilizers and manures, wastewater disposal and leaching from natural vegetation. Ammonia form of nitrogen from both animal and human wastes are oxidized to nitrite which is another alternative means in which nitrate leach/enters the groundwater aquifer or regime to contaminate them. In higher concentrations, nitrate may produce a disease called methemoglobinemia (blue baby syndrome) which affects bottle-fed infants (Jain et al. 2010). Constant ingestion of nitrate may also cause carcinogenic diseases (Jain et al. 2010). The physicochemical contaminants in the water samples from the study area are within the recommended limits by the WHO, SON and FME, but their presence in drinking water cannot be underestimated as further accumulations without treatment will adversely affect the health status of the inhabitants of the study area.

**Table 1:** Results of the physiochemical parameters of water samples from surface, shallow wells and municipal boreholes in the study area (rainy season)

	Parameters																							
Sample number	PH			Turbidity (FAU)			Electrical conductivity (μs/cm)			Total dissolved solid (mg/l)			Total hardness (mg/CaCO_3_)			Nitrate (mg/l)			Sulphate (mg/l)			Phosphate (mg/l)		
	S/W	R/W	B/H	S/W	R/W	B/H	S/W	R/W	B/H	S/W	R/W	B/H	S/W	R/W	B/H	S/W	R/W	B/H	S/W	R/W	B/H	S/W	R/W	B/H
AKO1	7.8	7.2	7.3	15.8	1.2	5.1	1100	361	980	0.2	0.3	0.3	47	96	110	4.0	3.0	4.1	6.8	7.2	7.0	2.0	1.0	1.0
AKO2	8.1	7.6	7.8	8.1	0.6	4.3	1150	930	1130	0.4	0.4	0.7	50	60	86	2.4	8.2	5.4	7.3	7.8	8.1	3.0	1.0	1.2
AKO3	8.3	7.3	7.4	11.1	0.8	4.4	1420	840	1650	0.2	1.0	1.8	64	50	70	3.3	4.7	8.6	5.1	23.1	14.1	3.0	1.0	1.2
EBE4	7.6	7.1	8.0	13.4	0.3	4.2	1320	640	1350	0.3	0.6	1.4	92	80	110	6.4	7.6	9.4	21.4	28.4	30.4	4.0	3.4	2.1
EBE5	7.8	7.4	7.3	26.2	1.8	2.8	1240	240	840	0.1	0.8	1.9	102	84	113	3.5	8.4	7.9	30.8	34.8	38.6	6.0	2.6	3.8
EBE6	8.4	7.1	8.4	18.4	2.4	4.6	1670	560	768	0.8	1.4	2.4	110	57	130	1.1	8.3	7.0	20.6	32.4	54.8	7.0	2.1	4.4
EZI7	7.3	7.3	7.4	16.8	2.9	4.8	1160	950	1120	0.8	0.8	3.4	86	60	86	2.8	6.3	8.5	38.5	26.1	40.4	9.0	2.6	2.6
EZI8	8.4	6.8	7.1	13.1	0.7	4.0	1340	870	1070	0.3	1.3	2.1	120	53	80	5.4	7.8	9.1	30.0	24.9	48.6	3.0	2.4	3.8
EZI9	8.3	7.1	6.5	21.0	0.4	1.3	1260	540	1170	0.9	2.6	1.4	109	62	90	5.1	8.1	8.0	35.4	18.4	34.6	2.0	1.4	3.4
UMU10	7.6	7.4	8.4	19.6	0.9	1.3	1110	810	1250	1.3	2.4	1.8	73	80	110	10.6	4.3	9.7	34.8	13.7	27.6	8.0	1.8	3.6
UMU11	6.2	6.6	7.9	18.4	1.7	0.6	1840	730	1200	2.6	4.1	2.6	84	64	78	9.1	5.9	8.7	18.4	18.4	36.4	1.0	2.4	4.7
UMU12	7.7	6.8	8.3	9.3	1.5	0.4	1170	620	1100	1.1	2.1	2.9	86	51	94	7.2	5.3	9.6	10.9	12.5	31.0	1.0	1.3	1.6
SON	6.5–8.5			5.0			1000			500			150			50			100			NS		
WHO	6.5–8.5			5.0			1.00			500			100			10			200			NS

**Table 2:** Results of the physiochemical parameters of water samples from the surface, shallow wells and municipal boreholes in the study area (dry season)

Sample number	PH			Turbidity (FAU)			Electrical Conductivity (μs/cm)			Total Dissolved Solid (mg/l)			Total Hardness (mg/CaCO_3_)			Nitrate (mg/l)			Sulphate (mg/l)			Phosphate (mg/l)		
	S/W	R/W	B/H	S/W	R/W	B/H	S/W	R/W	B/H	S/W	R/W	B/H	S/W	R/W	B/H	S/W	R/W	B/H	S/W	R/W	B/H	S/W	R/W	B/H
AKO1	6.4	6.8	7.1	13.9	0.6	3.1	1040	201	1010	0.5	0.2	0.8	44	91	105	3.8	3.1	3.7	6.6	7.1	5.7	2.0	0.7	1.1
AKO2	7.7	6.6	7.7	9.0	0.6	3.4	1070	730	1220	0.6	0.6	0.4	54	54	76	2.6	8.2	4.3	7.1	6.2	7.4	2.6	1.0	1.3
AKO3	8.1	7.1	7.8	12.9	0.8	4.7	1130	730	1560	0.1	1.3	1.5	60	46	70	3.1	4.4	9.1	4.6	20.3	12.9	2.0	0.9	1.2
EBE4	5.5	7.4	8.1	14.4	1.3	4.0	1430	740	1450	0.2	0.4	1.6	72	81	100	5.4	7.1	8.3	20.8	27.9	22.2	3.3	3.2	1.8
EBE5	6.7	7.8	7.8	24.2	1.4	3.7	1150	310	870	0.1	0.6	1.4	87	82	102	4.1	7.9	7.7	31.6	31.3	33.0	4.0	2.3	3.1
EBE6	8.1	7.9	8.4	20.6	2.2	4.5	1070	470	690	0.8	1.1	2.3	120	52	110	1.3	8.0	7.3	18.4	30.1	55.7	6.8	2.5	4.1
EZI7	6.8	8.3	7.1	17.8	1.9	4.1	1260	405	1320	0.7	1.8	4.4	96	53	76	1.8	5.9	6.4	35.7	22.6	38.2	7.7	2.3	2.2
EZI8	8.1	8.1	7.0	15.118.1	0.7	3.9	1340	910	1470	0.4	1.0	3.1	118	59	81	4.6	5.8	9.2	33.2	22.4	44.8	3.1	2.4	3.6
EZI9	8.5	6.8	6.9	17.6	0.5	1.5	1450	240	1380	0.8	2.2	1.2	104	71	87	4.8	8.4	7.4	34.6	20.6	33.9	1.8	1.2	2.9
UMU10	7.4	8.1	8.2	12.4	0.8	1.1	1010	860	1120	1.4	2.2	1.3	84	78	99	8.5	4.1	8.7	29.9	14.2	28.7	7.1	1.7	2.6
UMU11	7.3	7.6	7.9	10.4	1.3	0.8	1610	760	1230	2.2	3.8.1.6	2.1	74	67	87	9.0	4.6	6.8	20.4	16.4	35.4	1.0	2.9	3.4
UMU12	7.1	6.6	8.1		2.4	0.9	1090	720	670	1.4		2.4	86	46	90	8.1	4.9	8.9	8.9	9.4	30.5	1.3	1.4	1.7
SON	6.5–8.5			5.0			1000			500			150			50			100			NS		
WHO	6.5–8.5			5.0			1.00			500			100			10			200			NS		

### Results of the bacteriological analysis

Tables 3 and 4 show the total faecal coliform counts from the water samples in the area. The total coliform values obtained from surface water (river) range from 1.0 × 10^2^ to 6.3 × 10^3^ CFU/ml, shallow well water varies from 1.1 × 10^3^ to 4.4 × 10^3^ CFU/ml and municipal borehole water range from 3.9 × 10^2^ to 7.6 × 10^3^ CFU/ml. The value of faecal coliform varies from 1.1 × 10^2^ to 7.0 × 10^3^ CFU/ml in surface water, 1.3 × 10^2^ to 4.3 × 10^3^ CFU/ml in shallow wells water and 1.3 × 10^3^ to 6.8 × 10^2^ CFU/ml in municipal borehole water. The values of the total coliform and faecal coliform were lower in the borehole water, followed by surface water and then highest in the shallow well water most especially the uncovered wells. In all water intended for drinking or distribution systems, faecal coliform and total coliform must not be detectable in any 100-ml samples. Drinking water should not contain any microorganism known to be pathogenic, i.e. capable of causing disease or any bacteria indicative of faecal pollution (WHO). The bacteriological results show that water samples from the water resources contain considerable levels of *Escherichia coli* (*E*. *coli*). Their presence automatically reduces water quality. The reasons for the bio-contamination of the surface water could be attributed to the influx of allochthonous materials through run-off, discharge of chemical and waste products from the riparian zone, mowing near the river, poor cloud cover, sedimentation of suspended solids, particulate organic matter that have already settled on the river bed and flooding. The reason for the contamination of shallow well water by microorganisms as observed in the study area could be attributed to the uncovered and shallowness of the wells which allows easy movement of particles from the environments, poor sanitary conditions within the areas where the shallow wells are located, children and animals having free access the wells, method of obtaining water from wells, the use of an unclean bucket to draw water from the wells, construction of the wells around pit latrines and septic tanks, drainage and flooding of surface water into the unprotected wells and constructed shallow water wells. The reason for the presence of microbial contaminants in the municipal borehole water could be attributed to the rusting pipe which allows seepage of microbial contaminants into the borehole that affects the water quality. Microbial contaminants can as well rapidly pass through the unsaturated zone to the water table. Generally, groundwater that ought to be traditionally considered to be beneficial to human health contains harmful contaminants that have been introduced through precipitation which infiltrate municipal landfills and incorporate some of the countless chemicals such as household cleaners, paints and insecticides. They leak and infiltrate into an aquifer introducing chemicals into the underground. Any of the water sources used for drinking or domestic purposes should not contain any organism of faecal origin (Blonde 1977; Akeredolu 1991). Tables 3 and 4 show the ratios of faecal coliform (*E. coli*) to total coliform. The ratio varies from 0.02 to 10.77 for surface water, 0.093 to 2.39 for shallow well water and 0.06 to 12.50 for borehole water. The ratio of faecal coliform (*E. coli*) to total coliform (Tables 3 and 4) which is greater than four (4) suggests the pollution of human faecal origin (Geldreich and Kenner 1969). The ratio of less than four (4) suggests possible contamination from a mixture of human and animal sources (Beaudion and Litsky 1981; Okuofu et al. 1990). It therefore indicates that contamination of the water bodies in the study area to be of human origin and mixture of human and animal sources.

**Table 3:** Results of the total faecal coliform counts, faecal coliforms and ratios of faecal coliform/total coliform of the water samples from the surface, shallow wells and municipal boreholes in the study area (dry season)

Sample numbers	AKO 1	AKO 2	AKO 3	EBE 4	EBE 5	EBE 6	EZI 7	EZI 8	EZI 9	UMU 10	UMU 11	UMU 12		WHO	SON
Parameters															
Total coliform	6.2 × 10^2^	1.0 × 10^3^	4.2 × 10^2^	5.1 × 10^3^	1.0 × 10^3^	4.5 × 10^3^	1.0 × 10^2^	2.4 × 10^2^	4.1 × 10^2^	3.8 × 10^2^	4.4 × 10^2^	3.1 × 10^2^	Surface water	0	10
	3.9 × 10^2^	1.5 × 10^3^	6.6 × 10^3^	1.1 × 10^3^	5.0 × 10^3^	1.7 × 10^3^	4.3 × 10^2^	2.2 × 10^3^	1.6 × 10^3^	1.0 × 10^3^	2.1 × 10^3^	1.4 × 10^3^	Borehole		
	2.2 × 10^3^	1.6 × 10^3^	1.2 × 10^3^	4.2 × 10^3^	2.6 × 10^3^	1.1 × 10^3^	1.3 × 10^3^	2.1 × 10^3^	3.2 × 10^3^	2.8 × 10^3^	1.9 × 10^3^	1.1 × 10^3^	Shallow well		
Faecal coliform	4.0 × 10^3^	1.2 × 10^3^	1.4 × 10^3^	8.1 × 10^3^	2.5 × 10^2^	1.0 × 10^2^	1.7 × 10^2^	6.7 × 10^3^	1.0 × 10^3^	3.0 × 10^2^	2.1 × 10^2^	2.4 × 10^2^	Surface water	0	0
	5.0 × 10^3^	2.1 × 10^3^	3.8 × 10^3^	1.0 × 10^2^	6.6 × 10^2^	3.6 × 10^2^	1.9 × 10^2^	1.5 × 10^2^	1.1 × 10^2^	6.4 × 10^2^	2.2 × 10^2^	3.0 × 10^2^	Borehole		
	1.1 × 10^2^	3.2 × 10^3^	3.8 × 10^3^	5.3 × 10^2^	5.4 × 10^2^	4.5 × 10^2^	2.1 × 10^2^	3.2 × 10^2^	1.7 × 10^2^	7.1 × 10^2^	2.8 × 10^2^	1.2 × 10^2^	Shallow well		
Ratios; faecal coliform/total coliform	6.45	1.20	3.33	1.59	0.25	0.02	1.70	27.91	2.44	0.80	0.48	0.77	Surface water	< 4 animal contamination > 4 human contaminant	
	12.82	1.40	0.58	0.10	0.13	0.02	0.48	0.07	0.07	0.73	0.11	0.21	Borehole		
	0.05	2.00	3.17	0.09	0.21	0.41	0.44	0.15	0.05	0.64	0.15	0.11	Shallow well

**Table 4:** Results of the total faecal coliform counts, faecal coliforms and ratios of faecal coliform/total coliform of the water samples from the surface, shallow wells and municipal boreholes in the study area (rainy season)

Sample numbers	AKO 1	AKO 2	AKO 3	EBE 4	EBE 5	EBE 6	EZI 7	EZI 8	EZI 9	UMU 10	UMU 11	UMU 12		WHO	SO N
Parameters															
Total Coliform	6.5 × 10^2^	1.1 × 10^3^	5.0 × 10^2^	6.1 × 10^3^	1.0 × 10^3^	4.5 × 10^3^	2.0 × 10^2^	3.4 × 10^2^	3.8 × 10^2^	4.6 × 10^2^	5.6 × 10^2^	3.5 × 10^2^	Surface water	0	10
	4.8 × 10^2^	1.6 × 10^3^	7.6 × 10^3^	1.2 × 10^3^	6.0 × 10^3^	1.7 × 10^3^	4.4 × 10^2^	2.3 × 10^3^	1.7 × 10^3^	1.0 × 10^3^	2.2 × 10^3^	1.7 × 10^3^	Borehole		
	1.4 × 10^3^	1.8 × 10^3^	1.5 × 10^3^	4.4 × 10^3^	3.6 × 10^3^	1.4 × 10^3^	1.3 × 10^3^	2.6 × 10^3^	3.9 × 10^3^	3.9 × 10^3^	2.9 × 10^3^	2.1 × 10^3^	Shallow well		
Faecal Coliform	7.0 × 10^3^	1.6 × 10^3^	1.6 × 10^3^	9.1 × 10^3^	2.4 × 10^2^	1.1 × 10^2^	2.6 × 10^2^	8.7 × 10^3^	1.0 × 10^3^	3.2 × 10^2^	2.0 × 10^2^	3.3 × 10^2^	Surface water	0	0
	6.0 × 10^3^	2.0 × 10^3^	4.2 × 10^3^	1.2 × 10^2^	6.8 × 10^2^	3.7 × 10^2^	2.1 × 10^2^	1.7 × 10^2^	1.3 × 10^2^	7.3 × 10^2^	2.4 × 10^2^	3.1 × 10^2^	Borehole		
	1.3 × 10^2^	4.3 × 10^3^	4.0 × 10^3^	6.4 × 10^2^	6.1 × 10^2^	4.6 × 10^2^	2.0 × 10^2^	4.1 × 10^2^	2.0 × 10^2^	8.1 × 10^2^	3.2 × 10^2^	2.6 × 10^2^	Shallow well		
Ratios; faecal coliform/total coliform	10.77	1.45	0.03	1.49	0.24	0.02	1.30	25.59	2.63	0.70	0.36	0.09	Surface water	< 4 animal contamination > 4 human contaminant	
	12.50	0.80	0.06	0.10	0.11	0.22	0.48	0.07	0.08	0.73	0.11	0.18	Borehole		
	0.090.10	2.39	0.27	0.15	0.17	0.33	0.15	0.16	0.14	0.21	0.11	0.12	Shallow well		

### Statistical analysis and correlation

The physicochemical and bacteriological data were subjected to various statistical computations (Tables 5 and 6) to determine their intra-seasonal variability. The variance (*ẟ*^2^) and standard deviations (*ẟ*) were used to measure how the data vary around the mean (Mz); coefficient of variation (CV) was used to indicate the relative amount of variability in the distribution. The low values of *ẟ*^2^, *ẟ* and CV for the physiochemical and bacteriological parameters (Tables 5 and 6) imply that the data distributions are homogenous and close to each other. The confidence interval (CL) of 95% shows that the interval contains Mz, *ẟ*^2^ and *ẟ* values. Statistical tests—correlation coefficient, *t* test and chi-square test (***χ***^2^)—were computed. The computations were tested for significance at the 0.05 level of significance (*α* = 0.05). Correlation coefficient test was used to test whether there is a statistically significant linear relationship between the concentration levels of the contaminants in the dry and rainy seasons and to determine the strength and direction of their association at 5% level of significance. The tabulated value of ***χ***^2^ for 23 degrees of freedom at 5% level of significance is 35.172. The calculated ***χ***^2^ value is 0.5636 (Table 5). Since the calculated value of ***χ***^2^ (0.5636) is less than the tabulated value (35.172), it is not significant. The tabulated value of *t* for 23 degrees of freedom at 5% level of significance is 2.069. The calculated *t* value is 0.21034 (Table 5). Since the calculated value of *t* (0.21034) is less than the tabulated value (2.069), it is not significant. From the tests, it is concluded that the concentration levels of the measured physicochemical parameters in the dry and rainy seasons do not differ significantly. The calculated correlation coefficient (*r*) is 0.9990 (Table 5). It reveals a high degree of positive correlation. It is concluded that the concentration levels of the contaminants are independent of the change of season.

**Table 5:** Statistical analyses of the physiochemical parameters

Dry season						Rainy season					
Parameter	WR	Mz	*ð*^2^	*ð*	CV	CL	Mz	*ð*^2^	*ð*	CV	CL
pH	SW	7.31	0.69	0.83	11.30	± 0.47	7.79	0.38	0.59	7.60	± 0.34
	RW	7.43	0.36	0.60	8.08	± 0.34	7.10	0.10	0.30	3.90	± 0.20
	BH	7.68	0.25	0.50	6.47	± 0.28	7.65	0.31	0.56	7.31	± 0.32
Turbidity (FAU)	SB	15.50	17.20	4.15	26.7	± 2.35	15.93	25.05	5.01	31.41	± 2.83
	RW	1.21	0.40	0.63	52.2	± 0.36	1.27	0.61	0.78	61.50	± 0.44
	BH	2.98	1.99	1.41	47.5	± 0.80	3.15	2.86	1.69	53.70	± 0.96
Electrical conductivity (μs/cm)	SW	1220.83	34,707.64	186.3	15.26	± 105.40	1315	48,541.67	230.12	16.76	± 124.66
	RW	589.70	56,970.39	238.7	40.48	± 135.05	674.25	45,888.69	223.74	31.77	± 121.20
	BH	1165.83	82,624.31	287.44	24.70	± 162.63	113.67	48,463.22	229.93	19.40	± 124.56
Total dissolved solid (mg/l)	SW	0.77	0.36	0.60	78.1	± 0.34	0.75	0.45	0.67	89.7	± 0.38
	RW	1.40	0.94	0.97	69.1	± 0.55	1.48	1.16	1.08	72.50	± 0.61
	BH	1.90	1.10	1.04	55.3	± 0.60	1.89	0.71	0.84	44.60	± 048
Total hardness mg/CaCO_3_	SW	83.0	526.85	22.95	27.57	± 12.99	85.30	501.69	22.40	26.30	± 12.70
	RW	65.0	218.50	14.80	22.70	± 8.36	66.42	203.08	14.25	21.46	± 8.19
	BH	90.25	153.40	12.38	13.72	± 7.01	96.4	293.91	17.10	17.80	± 9.70
Nitrate (mg/l)	SW	4.76	6.09	2.47	51.86	± 1.40	5.08	7.34	2.71	53.40	± 1.53
	RW	6.03	3.10	1.76	29.20	± 1.00	6.49	3.11	1.77	27.18	± 1.00
	BH	7.32	2.96	1.72	23.50	± 0.97	8.00	2.73	1.65	20.60	± 0.93
Sulphate (mg/l)	SW	20.98	130.40	11.42	54.42	± 6.46	21.70	135.58	11.60	53.70	± 6.59
	RW	19.04	67.49	8.22	43.14	± 4.65	20.60	76.90	8.77	4250	± 4.96
	BH	29.03	203.80	14.28	49.17	± 8.08	30.92	205.20	14.30	46.30	± 8.10
Phosphate (mg/l)	SW	3.56	5.10	2.26	63.46	± 1.28	7.10	6.90	2.60	64.37	± 150
	RW	1.88	0.64	0.80	42.56	± 0.45	1.92	0.57	0.75	39.30	± 0.43
	BH	2.42	0.95	0.97	40.42	± 0.55	2.80	1.60	1.30	45.80	± 0.70

*t* test = 0.21034; chi-square test = 0.5636; *r* = 0.9990

*Mz*, mean; *ẟ*^2^, variance; *ẟ*, standard deviation; *CV*, coefficient of variation; *CL*, confidence level; *r*, Pearson correlation coefficient; *WR*, water resources

**Table 6:** Statistical analyses of the bacteriological parameters

	Dry season				Rainy season			
WR	Mz	*ẟ*^2^	*ẟ*	CL	Mz	*ẟ*^2^	*ẟ*	CL
SW	3.72	51.51	7.18	4.06	3.91	55.20	7.43	4.20
RW	1.29	11.49	3.39	1.92	1.39	12.02	3.47	1.96
BH	0.36	0.38	0.62	0.35	0.35	0.86	0.93	0.52

*t* test = 0.0546; chi-square = 0.9373; *r* = 0.9990

*Mz*, mean; *ẟ*^2^, variance; *ẟ*, standard deviation; *CL*, confidence level; *r*, Pearson correlation coefficient; *WR*, water resources

The tabulated value of ***χ***^2^ for 2 degrees of freedom at 5% level of significance is 5.991. The calculated ***χ***^2^ value is 0.9373 (Table 6). Since the calculated value of ***χ***^2^ (0.9373) is less than the tabulated value (5.991), it is not significant. The tabulated value of *t* for 2 degrees of freedom at 5% level of significance is 4.303. The calculated *t* value is 0.0546 (Table 6). Since the calculated value of *t* (0.0546) is less than the tabulated value (4.303), it is not significant. From the tests, it is concluded that the concentration levels of the measured bacteriological contaminants in the dry and rainy seasons do not differ significantly. The reasons could be attributed to high temperatures that could have killed some of the microorganisms, decomposition of some chemicals and effluents from surrounding due to flowing water into the sources. The calculated correlation coefficient (*r*) is 0.9990 (Table 6). It reveals a high degree of positive correlation. It is concluded that the concentration levels of the bacteriological contaminants are independent of intra-seasonal changes.

### Strategies for improving and supplying water of good quality in the study area

The use of waters from the study area for drinking, domestic and industrial purposes is problematic at their present conditions due to the presence of physicochemical and bacteriological contaminants and in most cases its high incidence. The inherent risk associated with this contamination is water-related diseases: waterborne diseases such as cholera, hepatitis, roundworm, diarrhoea, whipworm, poliomyelitis, typhoid and cryptosporidium (Ibrahim et al. 2000; Adeyinka et al. 2014; Nwabor et al. 2016); water-washed diseases such as trachoma, scabies, typhus and louse infestation; water-based diseases such as Guinea worm and schistosomiasis (Biu et al. 2009); diseases transmitted by water-related insect vectors such as yellow fever, filariasis, African trypanosomiasis, malaria, onchocerciasis, leishmaniasis and dengue (WHO 2005; UNICEF 2005). However, cryptosporidium as a protozoan may not be found in all African countries. Also, poliomyelitis may have been completely eradicated in some African countries but their reoccurrence cannot be ruled out if contaminated water is been continuously taken. Good quality water supplies to these communities are very essential in the protection of human health and well-being. Therefore, it becomes necessary to put forward the following strategies to improve the water quality of the study area. The hand-dug wells should be deep and properly covered, personal and environmental sanitary procedures should as well be adopted. Septic tank and open and pit latrines which are common in the area should be built far away from the surface, hand-dug well and municipal borehole waters. Solid waste dumpsites and petrol stations should be in approved places which must be far away from residential buildings. Otherwise, wells or boreholes dug close to pit latrines, septic tanks and petrol stations can be a serious source of contamination. Boiling and disinfection of water should be done before usage to prevent waterborne diseases. Contaminating sources should be controlled through modern and cheap sanitary engineering control programme not yet utilized. Bio-denitrification water treatments should be done for affected waters that use chemicals for agriculture before use. The application of fertilizers and pesticides in the soil most especially the farmlands and gardens that are close to homes and other residential areas should be prohibited or reduce lawn fertilizer and pesticide usage as when over-applied before a rainstorm, the chemicals run-off directly into the local waterways. Mowing near streams and ponds eliminates the natural contaminant protective buffers such as tall grasses, shrubs and trees. Mowing should be stopped near streams and ponds as natural buffers protect them against erosion, filter storm-water run-off reducing harmful pollutant loads and provide habitat for mosquito-eating amphibians, fish, birds and beneficial insects. Regular cleaning of storm drains and curbside that collects along nearby catch basins promotes cleaner run-off and reduces the amount of pollution and trash entering communities’ waterways. Testing and treating ponds are important in preventing harmful weeds and algae growth. It provides an array of eco-friendly approaches to control nuisance species and promotes the continual health of the water resources in the study area. A stewardship plan in the community focused on controlling invasive species and protecting the long health of open spaces, forests habitats, wetlands and water quality in the communities is developed. Exposure of soil to erosion can be reduced by planting native plants. Their roots stabilize the soil, reduce soil erosion and prevent sediment loading in the waterways which has huge impacts on the water quality of downstream rivers, ponds and reservoirs. Water supply legislation should serve as a policy instrument to address water quality standards. The effective and efficient surveillance system should be put in place to avoid indiscriminate dumping of waste products and refuse in the communities to protect the rural dwellers. Boiling water for 1 min inactivates all microbes and biological toxins. Municipal water treatment plants use standard series of steps: coagulation, sedimentation, filtration disinfection and storage. Of late, deadly Coronavirus (COVID-19) being faced globally is being traced to have originated from bats or pangolin as wild animals. The sampled sources show that water resources in the studied area were contaminated by humans and animals but no trace of particular domestic or wild animals. To avoid contamination by animals, there should be habitat modifications, the introduction of noisemakers’, predator decoys, scarecrows or plastic owls and the daily presence of human beings. These will keep the animals both domestic and wild animal species away from the sources of the available water resources. More so, the inhabitants of these communities should be well educated on the dangers posed by continuous drinking of contaminated water from these studied waters if not treated. The government should provide enough funds to water authorities to embark on actions that will expand the level of services and allow these communities to be served with water of good quality. The contractors and water managers should as well be sincere with the fund provided for that purpose such that the specification and better service delivery are met. Continuous monitoring is also required to prevent disease outbreak and public health burden in the area.

## Conclusion

The water resources of the western Niger Delta region were of poor quality on the bases of the presence of physicochemical and micro-bacteriological contaminants, odour, taste and colour. Ideally, clean water of good quality should not contain any physicochemical and bacteriological contaminants known to be pathogenic that is capable of causing diseases. Good quality water must be free from tastes, odour and colour. The inhabitants consume the contaminated water from these sources of water supply. The possible sources of the contaminants are likely natural and artificial sources of contaminants such as run-off from fertilized lands, septic tanks, industrial discharges, sewage and waste disposal, algae blooms and erosion from natural deposits. The health public implications include water-related diseases such as waterborne diseases, water-washed diseases, water-based diseases and diseases transmitted by water-related insect vectors. The remedies to improve and provide water of good quality in the study area protection through proper sanitation systems, limiting up-stream discharges, maintenance of wellheads, boiling and municipal water treatment plants and harassment activities that keep away animals from sources of water resources. Educational advice should be given to the inhabitants on the dangers posed by continuous drinking of contaminated water. These measures will prevent disease outbreak and public health burden in the area. The physicochemical and bacteriological data subjected to statistical and correlation tests show that their concentration levels were not dependent on intra-seasonal variations.
